# Metabolic small talk during exercise: The role of metabokines and lipokines in interorgan signalling

**DOI:** 10.1016/j.coemr.2024.100525

**Published:** 2024-06

**Authors:** Shaimaa A. Gad, Hannah Smith, Lee D. Roberts

**Affiliations:** 1 Leeds Institute of Cardiovascular and Metabolic Medicine, School of Medicine, University of Leeds, Leeds, UK; 2 Faculty of Medicine, Mansoura University, Egypt

**Keywords:** Metabokine, Lipokine, Exercise, Skeletal muscle, Interorgan signalling, Metabolite, Metabolism

## Abstract

Metabolites in exercise have traditionally been viewed as a fuel source, waste product, or anabolic components required for exercise-induced biosynthetic processes. However, it is now recognised that metabolites and lipids may act as mediators of interorgan crosstalk to coordinate the local and systemic physiological adaptations required to meet the complex system-wide challenge of exercise. These bioactive metabolite and lipid signals have been termed metabokines and lipokines, respectively. There is emerging evidence that metabokines and lipokines contribute to the health benefits of exercise. This review highlights several of the key recent discoveries related to metabokine and lipokine signalling during exercise. The discovery of these metabokines and lipokines, and their signalling targets, may provide the basis of future therapies for human disease.

## Introduction

Exercise is a highly regulated systemic physiological process that requires communication across multiple tissues and organ systems. During exercise training, multiple bouts of activity stress physiology, leading to whole-body adaptations from cells to tissues and organs [1]. Exercise is an established and effective intervention for the prevention or treatment of multiple pathologies and disease risk factors, such as obesity, type 2 diabetes, cardiovascular disease, and many cancers, and is also efficacious in limiting the age-related reduction in quality of life [2,3]. Given the plethora of health benefits, identifying the underlying molecular mechanisms through which exercise exerts its system-wide adaptive effects and the inter-organ messengers that facilitate this coordination have been the subject of intensive recent research [4,5]. This is a significant but important challenge with the potential to identify both therapeutic targets and new strategies to treat a range of diseases. Through these endeavours, many protein cytokine messengers released from exercising skeletal muscle, termed myokines, have been identified in studies of rodents and humans (reviewed in Refs. [4,6, 7, 8]). Several of these, such as irisin, meterorin-like myostatin and interleukin-6 (IL6) coordinate crosstalk between muscle and adipose tissue, establishing the importance of muscle-adipose tissue signalling axes in exercise [9, 10, 11, 12]. However, as we will establish, the tissue crosstalk functioning in exercise goes beyond the relationship between these two tissues [13].

From a metabolite-centric perspective, we can take adipose tissue – skeletal muscle crosstalk as an example. In exercise, the relationship between adipose tissue and skeletal muscle was regarded primarily from a viewpoint of fuel storage, selection, and availability. During aerobic exercise, mitochondrial fatty acid β-oxidation provides a key source of energy to maintain skeletal muscle contraction [14]. Fatty acids are released from triacylglycerol stores in adipose tissue via lipolysis and enter the circulation to provide fuel for contracting muscle. This is representative of a traditional viewpoint of metabolites in exercise, where they are often regarded as passive participants required either to maintain the energy demands of exercise (glucose, free fatty acids, ketones, amino acids) or as substrate for the biosynthetic processes initiated by exercise (e.g. amino acids in muscle hypertrophy). However, it is now recognised that metabolites may act as mediators of interorgan crosstalk to coordinate the local and systemic physiological adaptations required to meet the challenge of exercise. These bioactive metabolite signals have been termed metabokines [15]. Developing contemporaneously with this new appreciation of the complexity of tissue-crosstalk mediated by metabolites is the understanding that bioactive lipids also have a key role in the communication between cells, organs, and tissues. These bioactive lipids have been termed lipokines. Here, we will focus on the role of metabokines and lipokines in interorgan crosstalk in mediating the systemic adaptive responses and health benefits of exercise.

## A new perspective on familiar metabolites

**Figure 1:**
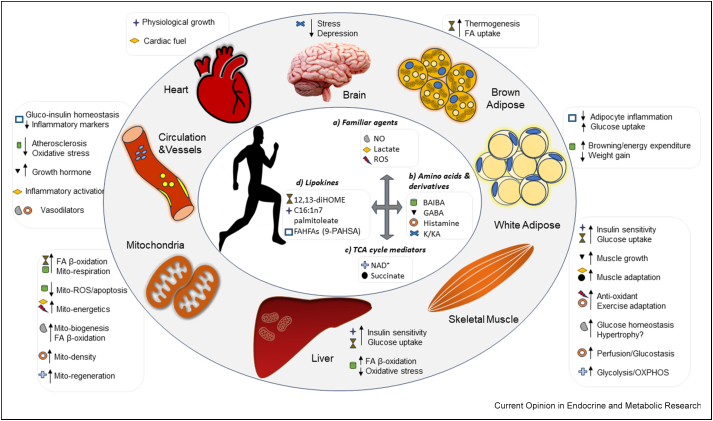
Schematic representation of metabokines involved in interorgan signalling during exercise, highlighting their target organs and metabolic effects. These metabokines include (**a**) metabolites commonly associated with exercise with emerging metabokine-like properties, (**b**) amino acids and their derivatives, (**c**) tricarboxylic acid cycle intermediates, and (**d**) lipokines. Metabokines are shape-coded against their metabolic effects in target tissues. K/KA, Kynurenine/Kynurenic Acid; FA, fatty acid; Mito, mitochondrial; ROS, reactive oxygen species; OXPHOS, oxidative phosphorylation.

There are few metabolites as intrinsically linked with exercise as lactate. Lactate is the end product of anaerobic glycolysis and historically it has been seen primarily as a waste product and fatigue agent accumulating in skeletal muscle during exercise-induced contraction. However, re-invigorated research interest in lactate over the last few decades has illuminated a role for this metabolite as an exercise-induced muscle-derived metabokine functioning through autocrine, paracrine, and endocrine mechanisms to regulate local and systemic physiology [16]. It is now clear that lactate has a role in the regulation of intermediary metabolism, mitochondrial energetics, skeletal muscle adaptation, redox biology, cardiac fuel selection, central nervous system function, appetite control, and inflammatory activation, amongst many more functions (Figure 1a) [17]. Beyond direct effects, lactate bioactivity can also result from the generation of downstream signals. During exercise, the lactate generated and secreted by skeletal muscle can be converted to N-lactoyl-phenylalanine (Lac-Phe) by monocytes, macrophages, and epithelial cells in humans and mice [18]. Lac-Phe then signals to the brain to suppress food intake and confer resistance to obesity [18]. The emergence of lactate as a metabokine has mirrored many discoveries in the field of metabokine research, where metabolites thought to be simple intermediaries, building blocks, or energy sources have been found to have much more complex roles in signalling and crosstalk.

Reactive oxygen species (e.g superoxide anion, hydroxyl radical and hydrogen peroxide), a set of metabolites that were previously viewed primarily as a by-product of mitochondrial dysfunction and mediators of oxidative damage, have now been found to have more nuanced roles in cellular signalling and the mediation of skeletal muscle adaptation to exercise. ROS are now understood to trigger downstream signalling pathways in skeletal myocytes to drive adaptive changes to maintain metabolic homeostasis during exercise through, amongst other mechanisms, activation of antioxidant systems and increased mitochondrial transport chain coupling efficiency (Figure 1a), reviewed in Ref. [19].

In contrast to lactate and ROS, nitric oxide (NO) has long been regarded as a pivotal signalling molecule since its discovery as a key regulator of vasodilation [20]. It is now known that NO production increases in skeletal muscle during exercise and drives mitochondrial biogenesis, fatty acid oxidation, regulates glucose homeostasis, and may have a role in hypertrophy (Figure 1a) [21,22]. Beyond these direct effects, NO has been suggested to regulate the expression of exercise-induced myokines, including IL6 [22]. Thus, it is clear that even for metabolites with established roles in skeletal muscle during exercise their function may include non-canonical signalling beyond their tissue of origin.

## Emerging exercise-regulated metabokines

The increased interest in metabolites as bioactive signalling molecules has led to the identification of metabokine signalling axes perpetrated by many novel metabolites and functioning across a distinct range of physiological processes, including the regulation of energy metabolism, immunity, tumour development, and central nervous system activity [15,23]. This is also true for auto-, para-, and endocrine signalling and interorgan communication during exercise.

### β-aminoisobutyric acid

The valine/thymine catabolite β-aminoisobutyric acid (BAIBA) was found to be released from skeletal muscle in response to aerobic exercise in mice and humans [24]. The synthesis and release of BAIBA from muscle are regulated by the transcriptional co-regulator, peroxisome proliferator-activated receptorγ co-activator 1α (PGC1α), a key regulator of the mitochondrial, metabolic, fibre-type, and angiogenic adaptive responses of skeletal muscle to exercise [24]. BAIBA signals in a muscle – adipose tissue – liver axis in which release from exercising muscle into circulation triggers the browning of white adipose tissue and enhanced fatty acid β-oxidation in the liver in mice through a PPARα-mediated mechanism [24]. These processes drive BAIBA-induced increases in whole-body energy expenditure, resistance to weight gain and enhanced glucose tolerance [24]. BAIBA has since been identified to signal through a skeletal muscle – bone axis during exercise, reducing mitochondrial ROS-driven apoptosis of osteocytes [25]. Moreover, BAIBA reduces hepatic and vascular oxidative stress [26,27], functions as an insulin sensitiser [28], reduces the development of atherosclerosis in mouse models [29], and enhances podocyte mitochondrial function [30]. Thus, BAIBA may contribute to many of the cardiometabolic health benefits of exercise (Figure 1b).

### Gamma-aminobutyric acid

Gamma-aminobutyric acid (GABA) is a non-protein amino acid and is canonically considered the primary inhibitory neurotransmitter in the central nervous system (CNS). However, it is also known to act outside the CNS with various non-neuronal properties in the cardiovascular system, pancreatic β-cells, cell proliferation and tumorigenesis, and the immune system [31]. During exercise in mice, GABA is released from skeletal muscle through a PGC1α-dependent mechanism [21,24]. GABA is enriched in the medium of transgenic PGC1α-expressing myocytes [24] and in the muscle and plasma of mice with muscle-specific transgenic expression of PGC1α (MCK-PGC1α) [21,32]. Moreover, concentrations of GABA are increased in the skeletal muscle and plasma of mice following 3 weeks of wheel running exercise [21]. This process was also determined to be regulated by the NO signalling pathway [21]. Long-term exercise training and plasma GABA increase the plasma concentration of growth hormone [33,34]. Interestingly, in a human study exploring a 12-week combined GABA and whey protein dietary supplement after resistance exercise, GABA further enhanced increases in whole-body fat-free mass when compared with protein supplementation alone [35]. Thus, GABA may also function as an exercise/PGC1α-mediated metabokine signal (Figure 1b).

### Kynurenine and kynurenic acid

The essential amino acid tryptophan is directly metabolised by the enzymes tryptophan 2,3-dioxygenase (TDO) and isoenzyme indoleamine 2,3-dioxygenases (IDO-1 and IDO-2) to form kynurenine. Subsequently, kynurenine is converted to kynurenic acid through the activity of kynurenine aminotransferases (KATs). During exercise, PGC-1α in skeletal muscle forms a transcriptional complex with PPARδ, which upregulates the expression of KATs, leading to increased production and secretion of kynurenic acid into circulation, and a subsequent reduction of the peripheral concentration of kynurenine in both rodents and humans [36,37]. This peripheral shift in equilibrium reduces the levels of kynurenine in the brain, reducing the stress-mediated effects that contribute to depression [36]. Therefore, kynurenine/kynurenic acid provides an interesting example of a metabokine-mediated skeletal muscle-brain signalling axis that may contribute to the effects of exercise on mood and behaviour (Figure 1b).

### Histamine

Acute and chronic aerobic exercise in humans triggers skeletal muscle and hepatic histamine release through mast cell degranulation or histidine decarboxylase (HDC)-mediated synthesis [38,39]. Recently, intramuscular temperature regulation of HDC was identified as a partial stimulus for histamine release during exercise [39,40]. An increase in intramuscular temperature to the exercise-induced maximum of 38.9 °C elicited a 41% increase in intramuscular histamine [39]. Subsequent histamine-mediated activation of H1 and H2 receptors in muscle promotes vasodilation and muscle glucose uptake, with the authors suggesting more research is required to establish whether this is via a direct role in enhanced insulin signalling [38]. The blockade of histamine H1/H2 receptors in humans attenuates exercise-induced improvements in glucose homeostasis, muscle perfusion, mitochondrial density, and mitochondrial antioxidant potential following a chronic exercise interval training programme [38]. Together, this suggests an autocrine/paracrine signalling role for histamine in the integrative adaptive response to exercise training through regulation of exercise capacity and skeletal muscle metabolic control (Figure 1b) [38].

### NAD+

Produced by either de novo synthesis or salvage pathways, nicotinamide adenine dinucleotide (NAD+), a critical energy intermediate in skeletal muscle glycolysis and oxidative phosphorylation, is elevated in muscle following exercise training in mice and humans [41,42]. Intriguingly, it was recently proposed that observed low NAD+/NADH ratios during acute high-intensity exercise trigger a sirtuin 1 (SIRT1)/CLOCK:basic helix-loop-helix ARNT-like protein 1 (BMAL1) feedback loop, prompting chronic adaptations to exercise training, including elevations in nicotinamide phosphoribosyltransferase (NAMPT), NAD+ salvaging, and NAD+/NADH ratios [42]. Accordingly, a recent study using rats found initial disruptions in skeletal muscle mitochondria and NAD+ after high-intensity aerobic exercise, along with increased protein levels of BMAL1, NAMPT, and SIRT1 [43]. Over time, these protein levels declined, NAD+ increased, and mitochondrial structure was restored, highlighting the importance of exercise and NAD+ release through circadian SIRT1/CLOCK:BMAL in regulating mitochondrial structure and function in exercise (Figure 1c) [43].

From an interorgan perspective, the liver is a key site of de novo NAD+ synthesis, where it is subsequently converted into nicotinamide, released into circulation (where hepatic nicotinamide accounts for ∼95% of the total in circulation in mice) and functions as a NAD+ precursor for skeletal muscle (and other organs) that rely primarily on the salvage pathway (reviewed in Ref. [42]). It has been postulated that exercise may modulate de novo NAD^+^ synthesis and nicotinamide release from the liver to accommodate increased demand by exercising skeletal muscle [42].

Additionally, since blunted NAD+ and exercise intolerance are common factors in multiple human diseases, recent NAD+ research has focused on developing NAD+ supplementation strategies to improve exercise outcomes [41,42]. The use of nicotinamide riboside (NR) supplementation in exercise performance and metabolic health has been a primary focus. In ANT1-deficient mice, which exhibit exercise intolerance, NR supplementation increased muscle NAD+ levels and improved exercise tolerance [44]. In patients with chronic kidney disease (CKD), who also demonstrate exercise intolerance, NR improved submaximal exercise capacity [45]. However, in both of these settings, NAD+ concentrations are depleted. Uncertainty remains regarding the effectiveness of NR to mediate exercise adaptation during NAD+ sufficiency [46].

### Succinate

The tricarboxylic acid cycle intermediate succinate has a distinct association with exercise, having first been observed to accumulate in the circulation of humans following bouts of exercise in the 1970s [47]. More recent data has suggested that succinate may function as a muscle-derived, exercise-regulated metabokine [48]. Succinate release from myocytes into both the interstitial fluid and blood was stimulated by exercise in humans and mice [48]. As myocyte acidification occurs during exercise, succinate becomes protonated and is transported out of the cells by the monocarboxylate transporter 1 [48]. Upon release from myocytes, succinate activates the succinate receptor SUCNR1 in non-myofibrillar cells, including stromal, satellite cells, and macrophages resulting in upregulation of axonogenesis and extracellular matrix remodelling programmes associated with the muscle adaptive response to exercise [48,49]. Mice lacking SUCNR1 fail to upregulate axonogenesis and extracellular matrix remodelling and exhibit impaired exercise training-induced increases in skeletal muscle strength [48]. Succinate is observed to mediate myocyte – stromal/satellite cell crosstalk to drive muscle remodelling, including myogenesis, and force transmission, and innervation in response to exercise stimulation (Figure 1c). This research provides an example of exercise-mediated metabokine signalling between cell types in a single tissue.

## Lipokines

There is increasing appreciation that bioactive lipids, known as lipokines, also play an important interorgan signalling role in exercise to contribute to the systemic adaptive responses and beneficial effects on health associated with exercise-mediated remodelling.

### C16:1n7 palmitoleate

In 2008, Cao et al. performed a murine study that identified the monosaturated n-7 fatty-acid C16:1n7 palmitoleate as an adipose-secreted lipokine, which increased insulin sensitivity in both the liver and skeletal muscle [50]. Independently, C16:1n7 palmitoleate has also been observed to enhance skeletal myocyte glucose uptake and hepatic insulin signalling [51,52]. During exercise, adipose tissue lipolysis is enhanced to maintain a supply of fatty acids to be used in skeletal muscle and cardiac fatty acid β-oxidation to meet energy demands. Simultaneously, exercise training induces cardiac remodelling, including left ventricular hypertrophy. Mice with impaired adipose tissue lipolysis through transgenic deletion of adipose triglyceride lipase exhibits not only blunted exercise-induced adipose tissue lipolysis but also reduced exercise-induced cardiac fatty acid uptake and cardiac hypertrophic remodelling [53]. This suggests that adipose tissue lipolysis promotes the development of exercise-induced cardiac hypertrophy. C16:1n7 palmitoleate was identified as a molecular co-mediator of exercise-induced cardiac hypertrophy functioning by inducing non-proliferative cardiomyocyte growth [53]. Palmitoleate provides an interesting example of an exercise-induced, lipokine-mediated, adipose tissue – cardiac tissue signalling pathway (Figure 1d). Although palmitoleate mediates insulin sensitivity and glucose uptake in muscle and liver, it remains to be seen whether the lipokine contributes to these processes in exercise.

### Fatty acid esters of hydroxy fatty acids

Mice with transgenic expression of Glut4 in adipose tissue exhibit increased lipogenesis and glucose tolerance [54]. Untargeted lipidomic analysis of the adipose tissue from these mice led to the identification of an enriched new class of lipids, branched fatty acid esters of hydroxyl fatty acids (FAHFAs) [54]. One particular species of FAHFAs, palmitic-acid-9-hydroxy-stearic-acid (9-PAHSA), was found to regulate glucose homeostasis, increase insulin sensitivity, and stimulate insulin secretion in mice [54]. In addition, PAHSA decreases adipocyte inflammation and enhances insulin-stimulated glucose uptake through a GPR120-mediated mechanism [54]. Recently, both acute aerobic exercise alone, and combined resistance and aerobic exercise training have been observed to stimulate the enrichment of FAHFAs in adipose tissue and in the circulation of humans [55,56]. This increase in exercise-stimulated FAHFAs corresponds with a decrease in inflammatory markers (Figure 1d) [56]. More research will be required to confirm whether FAHFAs contribute to the adaptive effects of exercise on fuel use and insulin sensitivity.

### 12,13-Dihydroxy-9Z-octadecenoic acid

12,13-Dihydroxy-9Z-octadecenoic acid (12,13-diHOME) is an oxylipin-class lipid derived from linoleic acid metabolism. This lipid was identified as a lipokine released from brown adipose tissue and enriched in the circulation of mice and humans in response to cold [57]. In murine studies, 12,13-diHome was found to stimulate brown adipose tissue thermogenic activity and increase cold tolerance [57]. 12,13-diHome increases brown adipose tissue fatty acid uptake by stimulating the translocation of the fatty acid transporters FATP1 and CD36 to the brown adipocyte plasma membrane [57]. Exercise stimulates 12,13-diHome production in and release from brown adipose tissue increasing the blood concentration of the lipokine in both mice and humans [58]. Further highlighting that not all exercise-mediated crosstalk originates from the skeletal muscle, exercise-stimulated BAT-derived 12,13-diHOME drives increased mitochondrial respiration, fatty acid uptake and fatty acid β-oxidation in skeletal muscle (Figure 1d); and therefore may support muscle fuel selection and use during aerobic exercise [58]. 12,13-diHOME operates in an exercise-mediated BAT-skeletal muscle signalling pathway.

## Summary

This review gives a brief overview of several of the key recent discoveries focussed on metabokine and lipokine signalling during exercise (Table 1). It is by no means exhaustive but will hopefully provide a starting point for greater exploration of the literature. We illustrate how the transition in perception of metabolites and lipids from passive participants in the metabolic process to bioactive signalling molecules has altered our view of their role in exercise. It is now clear that metabokines and lipokines have diverse functions in contributing to the adaptive response to exercise through signalling axes across multiple tissues and organ systems. It is also becoming increasingly apparent that metabokines and lipokines contribute to the health benefits of exercise. There is great promise that this new knowledge regarding metabokines and their signalling targets can be translated into novel future therapies for human pathology.

**Table 1:** Summary table outlining the origin and target tissues, downstream pathways activated, and resultant function of the exercise-induced metabokines/lipokines within the scope of this review.

Metabokine/Lipokine	Tissue of origin	Target tissue	Function	Downstream pathways
Lactate	Skeletal muscle [16] Liver [16] Integument [16]	White adipose tissue [16] Skeletal muscle [16] Liver [16] Kidneys [16] Cardiac [16]	↑Mitochondrial energetics [16] Cardiac fuel selection [17] Inflammatory activation [16] Skeletal muscle adaptation [16] Redox change [16] Regulation of intermediary metabolism [17]	HCAR-1 (adipose tissue, skeletal muscle) [16] Histone lacylation, PGC-1a, IGF-1 and sirtuin 1/3 [16] (metabolically active tissue) [16] TGF-β2 (adipose tissue) [16] Allosteric binding redox? (liver, kidneys) [16]
Reactive oxygen species (ROS)	Skeletal muscle [19]	Skeletal muscle [19]	Antioxidant activation [19] ↑Mitochondrial energetics [19]	Nrf2/KEAP NF-κB [19] FOXO transcription factors [19] Akt/mTORC1 [19] MAP kinases [19] AMPK [19] Calcium ion channels [19]
Nitric oxide (NO)	Skeletal muscle [22] Endothelium [20]	Skeletal muscle [21,22] Vasculature [20,22]	Mitochondrial biogenesis (skeletal muscle) [21] Increased fatty acid β-oxidation (skeletal muscle) [21] Glucose homeostatic regulation (skeletal muscle) [21] Skeletal muscle hypertrophy? [22] Vasodilation [20,22]	mTOR/p70S6K (skeletal muscle hypertrophy) [20] *IL-6* and *IL-*8 mRNA upregulation (skeletal muscle) [20] Guanylate cyclase/cGMP (vasodilation) [20]
β-aminoisobutyric acid (BAIBA)	Skeletal muscle [24]	White adipose tissue [24] Liver [24,26] Osteocytes [25] Vasculature [27,29]	White adipose tissue browning [24] ↑Hepatic β-oxidation [24] ↑Whole-body energy expenditure (white adipose tissue) [24] Resistance to weight gain (white adipose tissue) [24] ↓Oxidative stress (osteocytes [25], liver [26], vasculature [27]) ↓Atherosclerosis [29]	PPARα (white adipose tissue browning, hepatic β oxidation) [24] AMPK (reduced hepatic oxidative stress) [26] Mas-related G protein-coupled receptor type D (osteocytes) [25] PGC-1β-ERRα/PPAR-δ and PPAR-γ (reduced vasculature oxidative stress) [27]
Gamma-aminobutyric acid (GABA)	Skeletal muscle [21,24,35]	Skeletal muscle [21,24,32,35]	↑ Plasma [Growth Hormone] [21,33,34] ↑Protein synthesis [35] Skeletal muscle hypertrophy [35]	Growth hormone? [21,33,34]
Kynurenine and Kynurenic Acid	Skeletal muscle [36,37]	Brain [36]	↓Stress-induced depressive behaviour [36]	PGC-1α1-PPARα/δ-KAT → increased conversion of kynurenine to kynurenic acid [36]
Histamine	Mast cells (skeletal muscle, liver) or HDC synthesis (skeletal muscle, liver) [38,39]	Skeletal muscle [38] Endothelial cells [38] Vascular smooth muscle cells [38]	Vasodilation/skeletal muscle perfusion [38] ↑Glucose uptake/homeostasis [38] ↑Mitochondrial density [38] ↑Antioxidant potential [38]	H1/H2 receptors acting in autocrine and paracrine signalling pathways [38]
NAD^+^	Skeletal muscle [42]	Skeletal muscle [42] Liver [42]	↑Gluconeogenesis [42] Mitochondrial function restoration (skeletal muscle) [43] ↑Exercise tolerance [43] and submaximal exercise capacity [45]	Circadian SIRT/CLOCK:BMAL1 feedback loop (skeletal muscle) [42,43]
Succinate	Skeletal muscle [48]	Skeletal muscle non-myofibrillar cells (stromal, satellite) [48]	Muscle adaptation (axonogenesis/extracellular matrix remodelling [48]	Monocarboxylate transporter 1-SUCNR1 pathway (skeletal muscle) [48]
*C16:1n7 Palmitoleate*	Adipose tissue [50]	Liver [50] Skeletal muscle [50] Cardiac tissue [53]	↑Insulin sensitivity (skeletal muscle, liver) [50] Insulin signalling (liver) [51] Enhanced muscle glucose uptake [52] Cardiac hypertrophy [53]	Akt pathway [53]
Fatty acid esters of hydroxyl fatty acids	Adipose tissue [54]	Adipose tissue [54]	↑Glucose uptake [54] Gluco-insulin homeostasis [54] ↓ Adipocyte inflammation [54]	GPR120 signalling (↑glucose uptake, ↓inflammation) [54,56]
12,13-Dihydroxy-9Z-octadecenoic acid (12,13-diHOME)	Brown adipose tissue [57,58]	Brown adipose tissue [57] Skeletal muscle [58]	↑Insulin sensitivity (BAT) [57] ↑Glucose tolerance [57,58] ↑Thermogenesis (BAT) [57] ↑Fatty acid uptake and β-oxidation (BAT, skeletal muscle) [57,58] ↑Mitochondrial respiration (skeletal muscle) [58]	FATP1 and Cd36 translocation (increased fatty acid transport) (BAT, Skeletal muscle) [57,58]

## Credit author statement

Shaimaa Gad, Hannah Smith and Lee Roberts have equally contributed to:

- Conceptualisation.

- Literature search.

- Validation and visualisation.

- Writing, editing, review.

## Declaration of competing interest

The authors declare they have no conflict of interest.

## Data Availability

No data was used for the research described in the article.
